# Physiologic Response to the Pfizer-BioNTech COVID-19 Vaccine Measured Using Wearable Devices: Prospective Observational Study

**DOI:** 10.2196/28568

**Published:** 2021-08-04

**Authors:** Alexander G Hajduczok, Kara M DiJoseph, Brinnae Bent, Audrey K Thorp, Jon B Mullholand, Stuart A MacKay, Sabrina Barik, Jamie J Coleman, Catharine I Paules, Andrew Tinsley

**Affiliations:** 1 Division of Internal Medicine Department of Medicine Penn State Health Milton S. Hershey Medical Center Hershey, PA United States; 2 Department of Biomedical Engineering Duke University Durham, NC United States; 3 Department of Surgery Denver Health Medical Center Denver, CO United States; 4 Division of Infectious Diseases Department of Medicine Penn State Health Milton S. Hershey Medical Center Hershey, PA United States; 5 Division of Gastroenterology Department of Medicine Penn State Health Milton S. Hershey Medical Center Hershey, PA United States

**Keywords:** COVID-19, wearable devices, remote physiologic monitoring, heart rate, heart rate variability, respiratory rate, sleep, REM sleep, deep sleep, wearable, vaccine, monitoring, respiratory, physiological, cohort

## Abstract

**Background:**

The Pfizer-BioNTech COVID-19 vaccine uses a novel messenger RNA technology to elicit a protective immune response. Short-term physiologic responses to the vaccine have not been studied using wearable devices.

**Objective:**

We aim to characterize physiologic changes in response to COVID-19 vaccination in a small cohort of participants using a wearable device (WHOOP Strap 3.0). This is a proof of concept for using consumer-grade wearable devices to monitor response to COVID-19 vaccines.

**Methods:**

In this prospective observational study, physiologic data from 19 internal medicine residents at a single institution that received both doses of the Pfizer-BioNTech COVID-19 vaccine was collected using the WHOOP Strap 3.0. The primary outcomes were percent change from baseline in heart rate variability (HRV), resting heart rate (RHR), and respiratory rate (RR). Secondary outcomes were percent change from baseline in total, rapid eye movement, and deep sleep. Exploratory outcomes included local and systemic reactogenicity following each dose and prophylactic analgesic use.

**Results:**

In 19 individuals (mean age 28.8, SD 2.2 years; n=10, 53% female), HRV was decreased on day 1 following administration of the first vaccine dose (mean –13.44%, SD 13.62%) and second vaccine dose (mean –9.25%, SD 22.6%). RHR and RR showed no change from baseline after either vaccine dose. Sleep duration was increased up to 4 days post vaccination, after an initial decrease on day 1. Increased sleep duration prior to vaccination was associated with a greater change in HRV. Local and systemic reactogenicity was more severe after dose two.

**Conclusions:**

This is the first observational study of the physiologic response to any of the novel COVID-19 vaccines as measured using wearable devices. Using this relatively small healthy cohort, we provide evidence that HRV decreases in response to both vaccine doses, with no significant changes in RHR or RR. Sleep duration initially decreased following each dose with a subsequent increase thereafter. Future studies with a larger sample size and comparison to other inflammatory and immune biomarkers such as antibody response will be needed to determine the true utility of this type of continuous wearable monitoring in regards to vaccine responses. Our data raises the possibility that increased sleep prior to vaccination may impact physiologic responses and may be a modifiable way to increase vaccine response. These results may inform future studies using wearables for monitoring vaccine responses.

**Trial Registration:**

ClinicalTrials.gov NCT04304703; https://www.clinicaltrials.gov/ct2/show/NCT04304703

## Introduction

The COVID-19 pandemic has had a substantial global impact resulting in over 165 million infections and nearly 3.5 million deaths worldwide [1,2]. Vaccines are required to end the pandemic. The first vaccine to receive emergency use authorization for prevention of COVID-19 infection by the United States Food and Drug Administration was the BNT162b2 messenger RNA (mRNA) COVID-19 vaccine (Pfizer-BioNTech COVID vaccine) that encodes the spike protein of the SARS-CoV-2 virus [3,4]. Following preliminary studies with this mRNA vaccine showing neutralizing antibody response, a phase 3 randomized clinical trial demonstrated that the Pfizer-BioNTech vaccine was safe and had an efficacy of 95% in reducing risk of contracting COVID-19 compared to placebo [4-6].

An estimated 21% of US adults report using wearable devices that objectively measure physiologic parameters [7]. Although marketed for personal use, the widespread nature and convenience of these devices allows health care professionals to monitor physiologic changes in real time [8]. The WHOOP Strap 3.0 has been externally validated for tracking of heart rate variability (HRV), resting heart rate (RHR), respiratory rate (RR), and sleep stage duration [9]. HRV is determined by the subtle variation in the time between successive heart beats, thus HRV is a measure of the balance between the sympathetic and parasympathetic nervous system and their composite effects on heart rate [10].

A recent study using the WHOOP device was able to track physiologic changes, specifically an increase in nocturnal RR and decrease in HRV, in individuals who reported COVID-19 infection. These changes were noted 2 days before symptom onset in 20% of individuals and in 80% of the cohort after symptom onset [11]. Other studies have used HRV and RR measured by wearable devices to prospectively and retrospectively predict and identify COVID-19 infection (confirmed by positive testing) [12-14]. Therefore, we postulated that it would be possible to track an array of physiologic responses following COVID-19 vaccination. This wearable remote monitoring strategy could serve as a proof of concept and guide design of future studies of the physiologic and immune responses to vaccines.

## Methods

### Study Population

**Figure 1:**
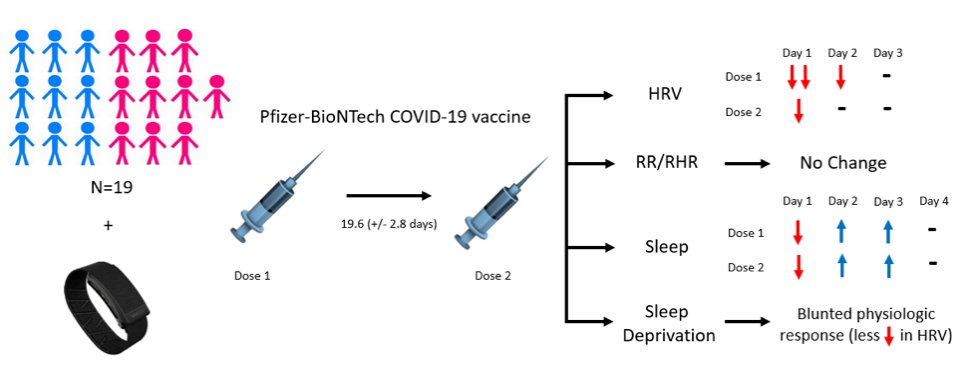
A total of 19 participants, 53% female, who were vaccinated with two doses of the Pfizer-BioNtech COVID-19 vaccine (mean time between doses 19.6, SD 2.8 days), transmitted continuous physiologic data via the WHOOP device. Changes from baseline were observed in HRV and were most pronounced on day 1 and 2 for dose 1 and only day 1 for dose 2. RR and RHR were unaffected following vaccination. Sleep duration initially decreased on day 1 post vaccine dose 1 and dose 2, with a compensatory increase from days 2 to 4, prior to return to baseline. Sleep deprivation was associated with a blunted HRV response, and premedication was associated with less change in RR and increases in REM and deep sleep percentages. HRV: heart rate variability; REM: rapid eye movement; RHR: resting heart rate; RR: respiratory rate.

Internal medicine residents at Penn State Hershey Medical Center previously enrolled in a clinical trial (NCT04304703) using the WHOOP Strap 3.0 to measure physiologic parameters were used for this analysis (Multimedia Appendix 1, Figure 1) [15].

### Study Design

The primary objective of this prospective observational study was to determine the physiologic changes following the first and second doses of the Pfizer-BioNTech COVID-19 vaccine. Primary outcomes were percent change from baseline in HRV, RR, and RHR for days 1 to 6 following each vaccine dose. Secondary outcomes were percent change from baseline duration of total, rapid eye movement (REM), and deep sleep. Exploratory outcomes included analysis of local and systemic reactogenicity (type and duration) associated with vaccination and prophylactic analgesic use.

### Study Procedures

Internal medicine residents were given a WHOOP Strap 3.0 to wear to measure physiologic parameters and sleep [15] (for full details, see Multimedia Appendix 2 [9,16-19]). Eligible participants were surveyed to disclose their vaccination dates for the novel Pfizer-BioNTech COVID-19 vaccine along with local and systemic reactogenicity and analgesic use following each vaccine dose.

Inclusion criteria for this analysis were patients concurrently enrolled in a clinical trial (NCT04304703) using the WHOOP device and who transmitted at least 80% of physiologic data during the study period including at least 24 of 45 days prior to dose one (to establish baseline metrics) and all data for the 6 days following vaccine dose one, dose two, or both. These data cutoffs were chosen based on published data using the WHOOP device for establishing a change from baseline in RR [11]. Local and systemic reactogenicity was graded as mild, moderate, or severe based on guidelines from the Centers for Disease Control and Prevention [1,20]. Patients were excluded if they did not or were unable to disclose the dates of vaccination (Multimedia Appendix 3). Data were blinded to study investigators for analysis. Recorded demographics included age, gender, comorbidities, and year of residency training.

Data collection was approved by the Institutional Board Review at Penn State Hershey Medical Center (STUDY14522).

### Statistical Analysis

We defined a significant change from baseline to be greater than 5% a priori. This cutoff was set based on recent findings in two studies: (1) changes in RR and other physiologic parameters in COVID-19–positive individuals, which were used to develop a predictive algorithm for COVID-19 infection risk stratification [11], and (2) precision measurements of heart rate, RR, HRV, and REM sleep stage duration using the WHOOP device were found to have less than 10% error [9].

The percent change of each metric for each participant in the data set was averaged together for the overall percent change of that metric for each day (d; equation 1). In equation 1, b_n_ is given as the average of the metric from the baseline period for participant n, and x_n_ is the value of the metric on the day (d) being calculated post vaccine dose.

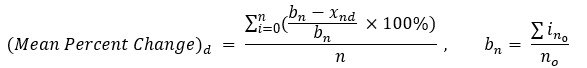



To determine the effect of sleep for the week leading up to the vaccine on the physiological effects of the vaccine, we computed Pearson correlations between hours of sleep in the 7 days prior to vaccine dose 1 and the percent changes of the physiological measurements post vaccine dose 1 [21].

Symptoms were aggregated and the density of the self-reported duration of symptoms was calculated [22]. Postvaccination reaction severity was compared to changes in physiologic parameters by Pearson correlations.

## Results

### Baseline Characteristics

A total of 19 participants met inclusion and exclusion criteria for this analysis; 18 individuals for dose 1 and 13 for dose 2 (Multimedia Appendix 3). Participants were 53% (n=10) female, with an age range of 26 to 35 years and a mean and median age of 28.8 (SD 2.2) and 29 years, respectively. No comorbidities were reported in 74% (n=14) of participants (Multimedia Appendix 1).

Baseline metrics were collected for all participants up to 45 days prior to vaccination dose 1 (Table 1). Mean baselines were as follows: RHR 63.09 (SD 6.36) bpm, HRV 52.09 (SD 21.58) ms, and RR 16.27 (SD 1.23) respirations per minute (rpm). Although interindividual variability in metrics had a wider range, intraindividual variability was much lower, most notably in nocturnal RR, with an intraindividual SD of mean 0.37 (SD 0.12) rpm.

**Table 1:** Baseline physiological and sleep metrics intraindividual mean and SD.

Metric	Intraindividual mean, mean (SD)	Intraindividual SD, mean (SD)
Heart rate variability (ms)	52.09 (21.58)	13.16 (6.94)
Resting heart rate (bpm)	63.09 (6.36)	4.95 (1.50)
Respiratory rate (rpm)	16.27 (1.23)	0.37 (0.12)
Sleep (hours)	6.71 (0.58)	1.48 (0.39)
REM^a^ sleep (%)	21.99 (6.71)	6.49 (1.23)
Deep sleep (%)	19.10 (2.15)	3.81 (0.66)

^a^REM: rapid eye movement.

### Physiologic Response to COVID-19 Vaccination by Dose

**Figure 2:**
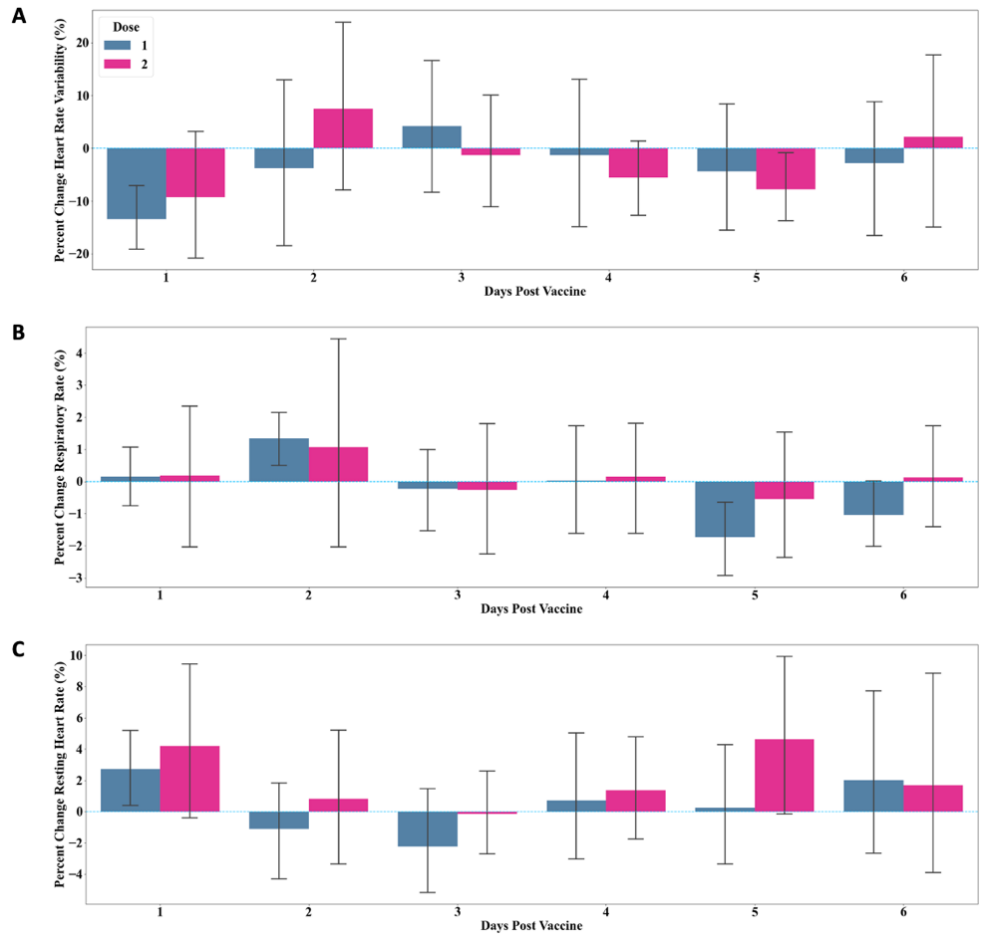
Percent change from baseline in (A) heart rate variability, (B) respiratory rate, and (C) resting heart rate, measured 6 days following COVID-19 vaccine dose 1 (blue) and 2 (magenta). Data is reported as mean (SD).

For dose 1 (n=18), there was a reduction in HRV on day 1 (mean percent change –13.44%, SD 13.62%). HRV returned to baseline by day 3 and remained at baseline thereafter (Figure 2A, blue; Table 2). There was no significant change in RHR and RR compared to baseline in the 6 days following vaccination (Figure 2B, C, blue; Table 2).

For dose 2 (n=13), HRV decreased on day 1 (mean percent change –9.25%, SD 22.69%) but quickly normalized to baseline by day 2 (Figure 2A, magenta; Table 3). Similar to dose 1, there was no significant change in RHR and RR in response to dose 2, with both metrics remaining at baseline from day 1 to day 6 (Table 3).

**Table 2:** Percent changes from baseline in physiological and sleep metrics for 6 days postvaccine dose 1 (n=18).

Metrics	Day 1, mean percent change (SD)	Day 2, mean percent change (SD)	Day 3, mean percent change (SD)	Day 4, mean percent change (SD)	Day 5, mean percent change (SD)	Day 6, mean percent change (SD)
Heart rate variability	–13.44 (13.62)	–3.74 (34.63)	4.21 (27.23)	–1.32 (30.39)	–4.35 (26.79)	–2.80 (27.46)
Resting heart rate	2.73 (5.50)	–1.10 (6.93)	–2.23 (7.31)	0.72 (8.80)	0.26 (8.68)	2.02 (11.48)
Respiratory rate	0.16 (1.95)	1.34 (1.98)	–0.23 (2.70)	0.02 (3.81)	–1.73 (2.56)	–1.04 (2.29)
Hours of sleep	–8.41 (22.96)	5.00 (18.27)	9.41 (21.60)	7.74 (17.81)	3.21 (24.38)	–3.21 (27.54)
Percent of REM^a^ sleep	–4.94 (37.65)	–6.53 (30.06)	–5.13 (33.34)	–6.70 (19.62)	–2.70 (31.10)	–16.98 (29.41)
Percent of deep sleep	9.64 (26.30)	3.08 (23.00)	4.11 (12.66)	4.58 (15.28)	–6.05 (19.58)	–2.38 (17.53)

^a^REM: rapid eye movement.

**Table 3:** Percent changes from baseline in physiological and sleep metrics for 6 days postvaccine dose 2 (n=13).

Metrics	Day 1, mean percent change (SD)	Day 2, mean percent change (SD)	Day 3, mean percent change (SD)	Day 4, mean percent change (SD)	Day 5, mean percent change (SD)	Day 6, mean percent change (SD)
Heart rate variability	–9.25 (22.69)	7.48 (32.44)	–1.30 (19.44)	–5.56 (13.70)	–7.76 (13.12)	2.19 (30.22)
Resting heart rate	4.20 (9.42)	0.82 (8.27)	–0.15 (5.15)	1.37 (6.17)	4.63 (10.38)	1.70 (12.83)
Respiratory rate	0.19 (4.10)	1.07 (6.44)	–0.26 (4.00)	0.15 (3.22)	–0.54 (3.63)	0.13 (3.06)
Hours of sleep	–2.10 (26.18)	5.33 (17.71)	6.06 (23.84)	9.22 (28.37)	–4.58 (20.45)	0.49 (12.30)
Percent of REM^a^ sleep	13.79 (45.88)	–8.73 (39.57)	12.67 (36.41)	0.64 (37.99)	6.12 (32.02)	–8.35 (43.06)
Percent of deep sleep	4.00 (25.02)	–1.70 (19.21)	3.56 (20.31)	–6.01 (22.09)	3.42 (21.31)	–11.37 (24.40)

^a^REM: rapid eye movement.

### Postvaccination Changes in Sleep

**Figure 3:**
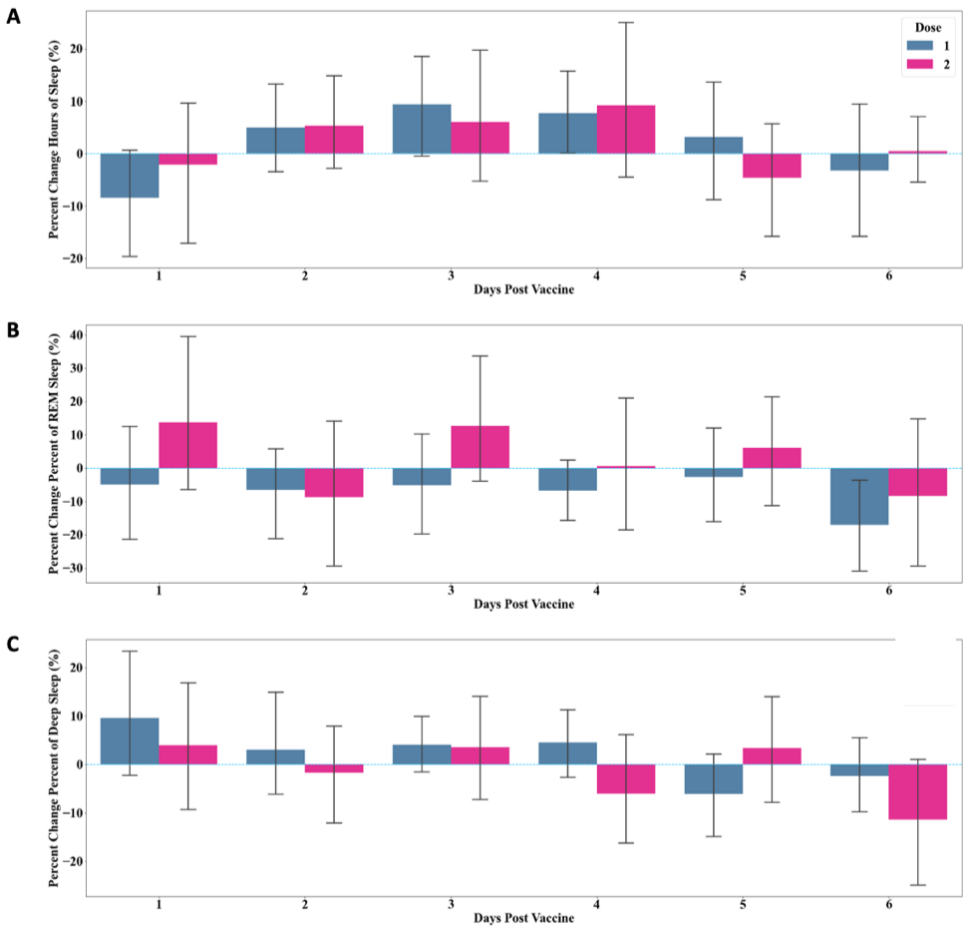
Percent change from baseline in (A) total sleep duration, (B) REM sleep duration, and (C) deep sleep duration, measured 6 days following COVID-19 vaccine dose 1 (blue) and 2 (magenta). Data is reported as mean (SD). REM: rapid eye movement.

Total sleep duration, REM, and deep sleep duration (in hours) were measured for all participants for 6 days following vaccine administration. Total sleep duration followed a similar overall pattern for both vaccine doses: an initial decrease was observed on day 1 (dose 1: mean –8.41%, SD 22.96%; dose 2: mean –2.1%, SD 26.8%) followed by an increase above baseline on days 2, 3, and 4, with subsequent return to baseline on days 5 to 6 (Figure 3A; Tables 2 and 3). The change in sleep duration peaked on day 3 following dose 1 and day 4 following dose 2. Total sleep duration was proportional to time in bed and thus showed similar trends in response to vaccine dose 1 and dose 2.

Patterns of change in REM and deep sleep did not follow the same pattern as total sleep duration but showed greater variability overall (Figure 3B, C; Tables 2 and 3). Total sleep cycles and sleep disturbances had no correlation with changes in physiologic metrics following either vaccine dose.

### Sleep Impact on HRV Changes

Sleep duration was evaluated 7 days preceding vaccine administration to establish an individualized baseline. During the baseline assessment period, participants slept, on average, 6 hours and 43 (SD 35) minutes per night, of which 21.99% (1 hour and 28 minutes) was REM sleep and 19.1% (1 hour and 17 minutes) was deep sleep. A greater amount of sleep in the 7 days prior to receiving the first dose of the vaccine was moderately correlated with a higher percent change in HRV the 2 days following vaccine dose 1 (Pearson *R*=0.570 day 1; *R*=0.494 day 2).

### Postvaccination Symptoms

**Figure 4:**
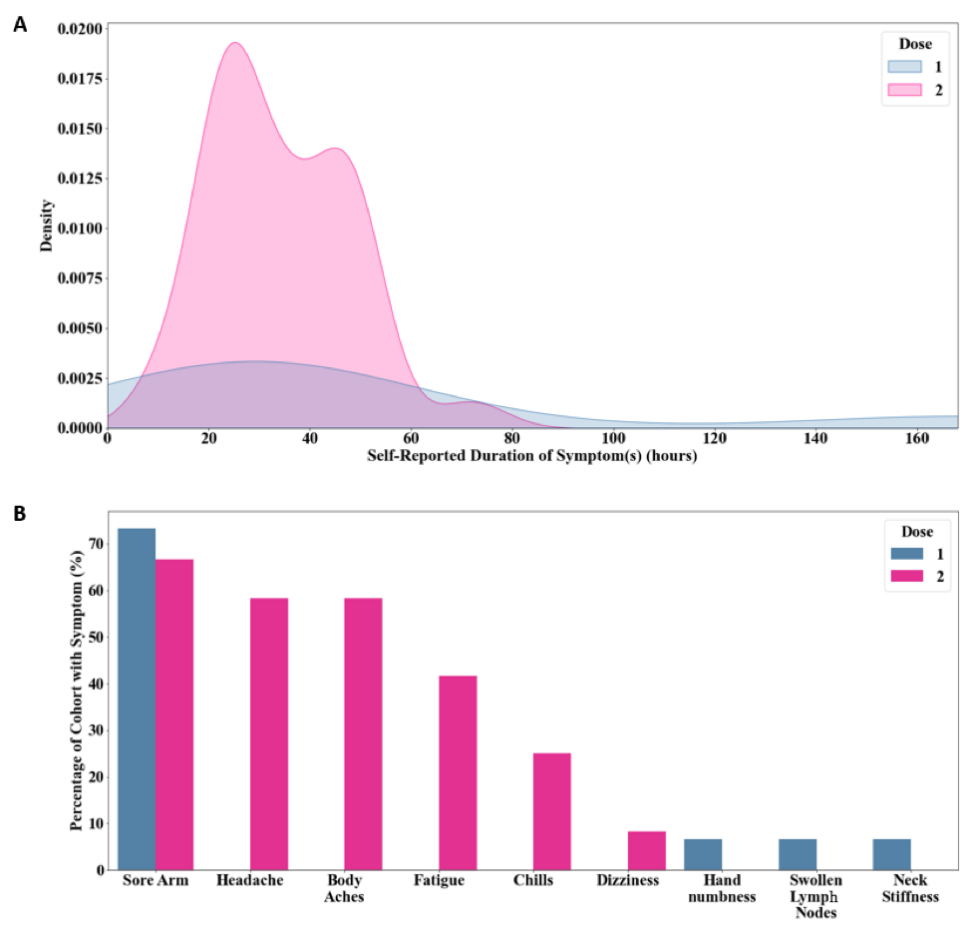
(A) Self-reported symptom duration following dose 1 and dose 2 of the COVID-19 vaccine. (B) Local and systemic reactogenicity experienced by participants by vaccine dose.

An array of local and systemic reactions to vaccination were reported by participants, ranging from arm soreness to fatigue and body aches. A greater frequency and duration of symptoms were reported following dose 2 (Figure 4A, B). Arm soreness was reported in more than 60% of participants for both doses. The majority of symptoms subsided by hour 60 post vaccination (Figure 4A). The mean symptom duration following dose 1 was 49.7 (SD 49.2) hours, which decreased to 34.1 (SD 13.3) hours for dose 2. The most frequent symptom duration after dose 1 and dose 2 was 24 hours. Overall, postvaccination reactogenicity would be classified as mild to moderate, as no severe adverse reactions such as angioedema or other allergic reactions requiring urgent treatment were reported [1,20]. Presence of postvaccination reactogenicity did not show a strong correlation with changes in sleep or other physiologic parameters.

### Analgesic Effects on HRV, Sleep, and Postvaccination Symptoms

None of the 19 participants premedicated with analgesic medications (ibuprofen or acetaminophen) prior to dose 1; however, 7 of the 13 (54%) participants premedicated prior to dose 2 (Multimedia Appendices 4 and 5). Overall changes in HRV were the same in both groups (premedication vs no premedication; Multimedia Appendix 5). Those who did not premedicate had a greater response (increase) in RR on day 1 and day 2, but overall RR was unaffected when both groups were analyzed together (Multimedia Appendix 4). RHR had a slightly greater increase on day 1 for those who did premedicate (Multimedia Appendix 4). The group that premedicated had both a greater initial decrease and compensatory increase in total sleep duration (Multimedia Appendix 5). This group also had higher percentage of REM and deep sleep in the days after receiving dose 2, which were most prominent on day 1 (Multimedia Appendix 5).

The duration of all reported symptoms between the groups were similar: participants without medication experienced symptoms for a mean of 30.0 (SD 13.4) hours, and participants who self-medicated prior to dose 2 experienced symptoms for 37.7 (SD 10.0) hours. There was no significant difference in symptom severity among the two groups [1,20].

## Discussion

### Principal Findings

In this small observational study in a relatively young and healthy cohort of participants, we provide evidence that consumer-grade wearable devices can be used to measure physiologic response to COVID-19 vaccination (Figure 1). HRV change from baseline was the most prominent signal in our study population, while RHR and RR were unaffected (Figure 2).

**Figure 5:**
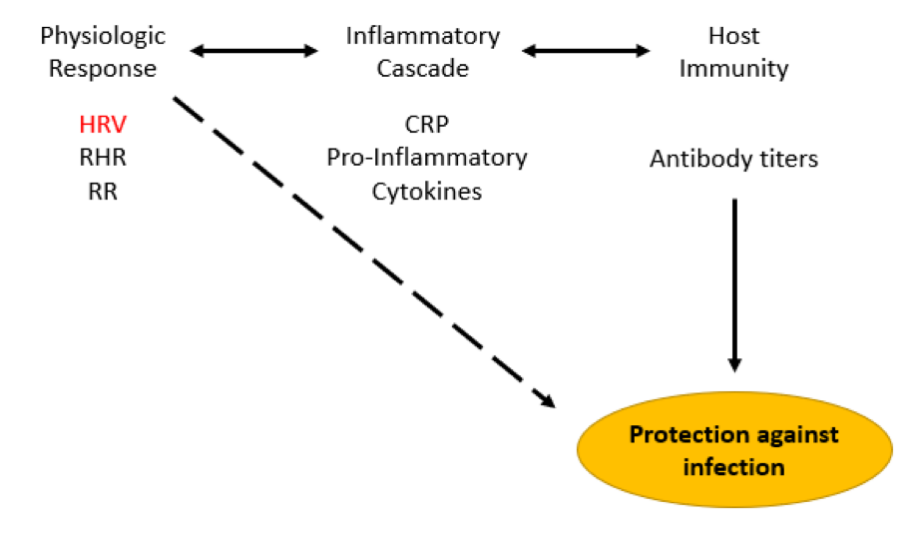
Hypothetical connection between physiologic response measured by wearables (HRV, RHR, RR), inflammatory response (serum CRP and proinflammatory cytokine levels), and host immunity dictated by antibody response to vaccination. Wearable monitoring of physiologic metrics could potentially be a simple and effective way to track the efficacy of vaccine-mediated protection against infection (dashed arrow). HRV highlighted as this parameter showed the greatest changes in this study. CRP: C-reactive protein; HRV: heart rate variability; RHR: resting heart rate; RR: respiratory rate.

Decreases in HRV, a surrogate of autonomic tone, have been shown to predict negative clinical outcomes following severe infections [23-25]. HRV decreases have also been correlated with an increased C-reactive protein (CRP) within the first 2 days following administration of the influenza A vaccine [26]. Lower magnitude CRP elevations have been associated with increased risk of infection, suggesting that greater HRV decreases and CRP increases would equate to a protective inflammatory or immunologic response [26,27]. Thus, we propose that a higher percent change in HRV may equate to a more robust immune response to COVID-19 vaccination [14,26,28-30]. The directionality of HRV change (decrease) is significant, as this is suggestive of increased parasympathetic tone due to generation of an immune response to the vaccine [14,28]. Further investigation of HRV response to vaccination could provide a useful surrogate marker for immune system activation (Figure 5) [31]. This could be accomplished in a randomized controlled trial (RCT) of vaccination versus placebo with wearable tracking of HRV in comparison to CRP levels, antibody titers, and protection against infection.

There was relatively no change in both RR and RHR in response to vaccination (Figure 2B, C). This is of particular interest given that spikes in RR are clinically relevant in prediction of COVID-19 infection and progression of disease [1,11-13]. Although we did not directly collect data on oxygen saturation or hypoxia, it is likely that changes in RR are specific to COVID-19 infection, which has a predilection for pulmonary pathology. Thus, this change would not be expected with vaccination as, unlike natural disease, it does not impact pulmonary function.

Interestingly, there was a moderate correlation between change in HRV and amount of sleep prior to vaccination (greater sleep was associated with a greater decrease in HRV). Sleep deprivation is known to have a significant impact on viral susceptibility and blunted adaptive immune response in the presence of viral vaccines [32-38]. Decreased antibody titers and overall immune response have been observed in vaccinated participants that are sleep deprived, most notably in response to the influenza and hepatitis A vaccine [34-39]. A recent study of 2884 health care workers showed that 1-hour longer sleep duration was associated with 12% lower odds of COVID-19 infection [40]. Our data demonstrate that sleep duration impacts physiologic response to COVID-19 vaccination and, if correlated with immune response in further studies, could be leveraged to potentiate the effectiveness of vaccination in general.

### Limitations and Future Studies

This is a small observational study in a specific cohort of participants with no control arm. A larger powered study is needed for formal statistical analysis of physiologic changes and to control for baseline demographics. The lack of a control group institutes bias; an RCT with a placebo arm (no vaccine) would allow for comparison of physiologic metrics to a control group. However, our use of *percent change from baseline* in our outcomes would help overcome intraindividual variability confounding of results (ie, individuals may have a greater magnitude change in parameters simply because they have a higher baseline, which is accounted for by using percent change from established baseline). Last, this population is known to have a greater degree of sleep deprivation secondary to duty hours and clinical demands, which may be a confounder and reduce the generalizability of the results [41].

Incorporation of biomarkers such as CRP is needed to corroborate association with physiologic changes (Figure 5) as previously observed in other vaccine studies [26,29,42,43]. Despite only exploring the response to the Pfizer-BioNTech COVID-19 vaccine, this study further confirms the feasibility of using wearable remote physiologic data to monitor responses to vaccines. This simple method to track vaccine responses would be useful for future novel vaccines. The key will be to determine how well remotely monitored physiologic metrics can predict inflammatory response, vaccination antibody titers, and ultimately protection from infection. This may provide a noninvasive method for individualized prediction of vaccine efficacy.

### Conclusion

Wearable devices are now widely available to everyday consumers, and as technology has advanced, they are being more widely used to capture medical data [7,8,12,13,44]. This study is a proof of concept for this remote monitoring strategy to capture physiologic response to COVID-19 vaccines. If correlated with immune response and vaccine efficacy in future studies, this approach could be leveraged in the general population to predict response to vaccines. Our data also raises the possibility that increased sleep prior to vaccination may impact physiologic responses. This warrants further study and is a potentially modifiable factor to optimize vaccine response.

## Appendix

Multimedia Appendix 1 Baseline characteristics of study participants.

Multimedia Appendix 2 Description of physiologic metrics.

Multimedia Appendix 3 Study flowchart. A total of 38 participants were initially enrolled in the study; 33 were screened for inclusion and exclusion criteria, yielding 19 participants that were included for final analysis (18 participants for dose 1 and 13 participants for dose 2).

Multimedia Appendix 4 Premedication subgroup analysis. Percent change from baseline in (A) heart rate variability, (B) respiratory rate, and (C) resting heart rate, measured 6 days following COVID-19 vaccine dose 2 is reported for participants who premedicated prior to vaccination (purple) and participants who did not premedicate (green). Data is reported as mean (SD).

Multimedia Appendix 5 Premedication subgroup analysis. Percent change from baseline in (A) total sleep duration, (B) rapid eye movement sleep duration, and (C) deep sleep duration, measured 6 days following COVID-19 vaccine dose 2 is reported for participants who premedicated prior to vaccination (purple) and participants who did not premedicate (green). Data is reported as mean and SD.

## Abbreviations

CRP: C-reactive protein
HRV: heart rate variability
mRNA: messenger RNA
RCT: randomized controlled trial
REM: rapid eye movement
RHR: resting heart rate
rpm: respirations per minute
RR: respiratory rate
