# Supplementary material for: Physiologic Response to the Pfizer-BioNTech COVID-19 Vaccine Measured Using Wearable Devices: Prospective Observational Study
Source: JMIR Form Res. 2021 Aug 4;5(8):e28568. doi: 10.2196/28568 (PMC8341091; doi:10.2196/28568)
Supplement: Multimedia Appendix 1 [file formative_v5i8e28568_app1.docx]

| Number of Subjects | 19 |
| --- | --- |
| Age (years) | 28.8 (+/- 2.2) |
| Gender (%) |  |
| Female | 10 (53%) |
| Male | 9 (47%) |
| Comorbidities (%) |  |
| None | 14 (74%) |
| Anxiety or Depression | 4 (21%) |
| Other^1^ | 3 (16%) |
| Year in Training (%) |  |
| PGY-1 | 10 (53%) |
| PGY-2 | 6 (31%) |
| PGY-3 | 3 (16%) |

^1^Obstructive sleep apnea (1), Attention Deficit Hyperactivity Disorder (1), Sarcoidosis (1)

**Supplementary Table 1:** Baseline Characteristics of study participants.
