# Supplementary material for: Physiologic Response to the Pfizer-BioNTech COVID-19 Vaccine Measured Using Wearable Devices: Prospective Observational Study
Source: JMIR Form Res. 2021 Aug 4;5(8):e28568. doi: 10.2196/28568 (PMC8341091; doi:10.2196/28568)
Supplement: Multimedia Appendix 2 [file formative_v5i8e28568_app2.docx]

**Appendix:**

***Physiologic Metrics***

The WHOOP Strap 3.0 is a wearable, waterproof, and rechargeable device containing a photoplethysmogram, accelerometer, capacitive touch sensor, and gyroscope that can be worn 24-hours per day, primarily on either wrist (1 cm above wrist bone, with sensor pressed against skin), lasts 5 days between charges, and wirelessly transfers data to mobile devices running the associated WHOOP application.

The WHOOP Strap 3.0 has been externally validated for tracking of HRV, RHR, RR and sleep stage duration [9]. Heart rate variability (HRV), a measure of autonomic tone, or the balance of the effects of the sympathetic and parasympathetic nervous system on the cardiovascular system, is measured by root mean square standard deviation (RMSSD) of successive heart beats (R-R interval difference) during the last 5 minutes of the last cycle of deep sleep [42]. This has been shown to be a standardized and reproducible time frame for measurement that minimizes intrinsic and extrinsic stimuli that can influence and distort HRV readings [43].

Resting heart rate (RHR) is defined as the measure of average heart beats per minute during complete rest, and is also measured during the last 5 minutes of the last cycle of deep sleep. Respiratory Rate (RR) is defined as the median value of respirations per minute and is derived each night during the main sleep period via photoplethysmography. This is based on respiratory sinus arrhythmia, or the variability in heart rate in synchrony with respiration, by which the R-R interval on an ECG is shortened during inspiration and prolonged during expiration [44].

Sleep is characterized by generalized cardiovascular activation and baroreflex sensitivity [45]. Sleep stages including rapid eye movement (REM) and non-REM phases, the latter of which can be broken down further to deep (also referred to as slow wave sleep; SWS) or light sleep. Both REM and deep sleep are considered restorative sleep, in which the autonomic nervous system dictates both sympathetic and parasympathetic nerve activity to allow for physical and mental recovery [43]. REM sleep exhibits high muscle and lumbar sympathetic nerve activity, sometimes exceeding sympathetic nerve activity seen during wakefulness with burst of elevated blood pressure and heart rate. Conversely, deep sleep is more stable with reduced blood pressure variability, constant autonomic activity, lower cardiac output and regular minute ventilation (respiratory rate x tidal volume), offering a highly standardized condition for reproducible HRV assessment [45]. Measuring HRV during deep sleep has been identified as the most reliable measurement period, devoid of both external (environmental) and internal (emotional) factors that can affect autonomic tone, and therefore HRV [43]. The WHOOP device samples the entirety of sleep duration in individuals to determine sleep stages based on HR and HRV changes, and has been shown to accurately predict sleep stages when tested against the gold standard of polysomnography and continuous electrocardiogram [9].
