# Supplementary figures and images for: Physiologic Response to the Pfizer-BioNTech COVID-19 Vaccine Measured Using Wearable Devices: Prospective Observational Study
Source: JMIR Form Res. 2021 Aug 4;5(8):e28568. doi: 10.2196/28568 (PMC8341091; doi:10.2196/28568)

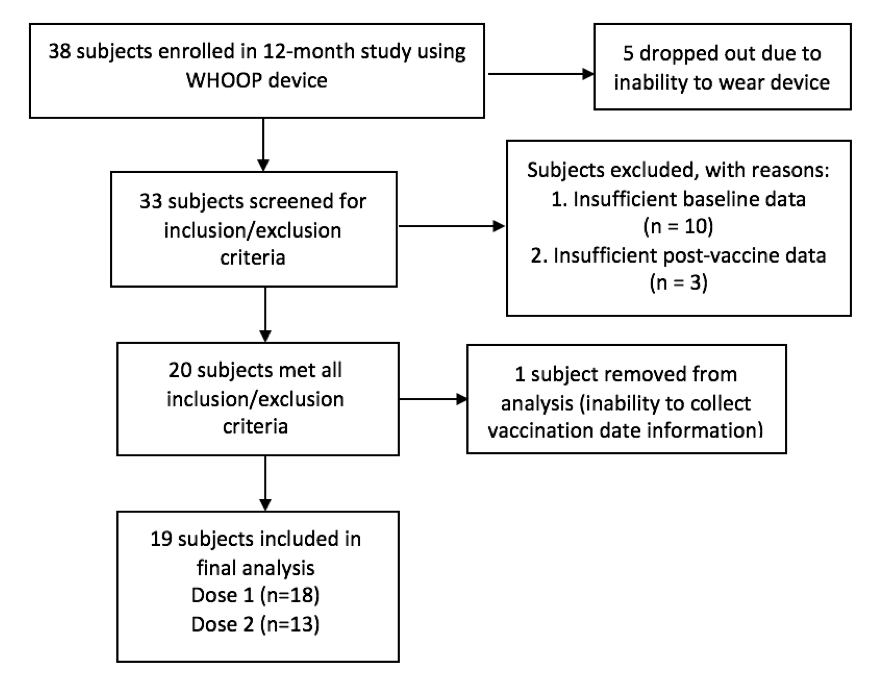

Supplement: Multimedia Appendix 3 [file formative_v5i8e28568_app3.png]

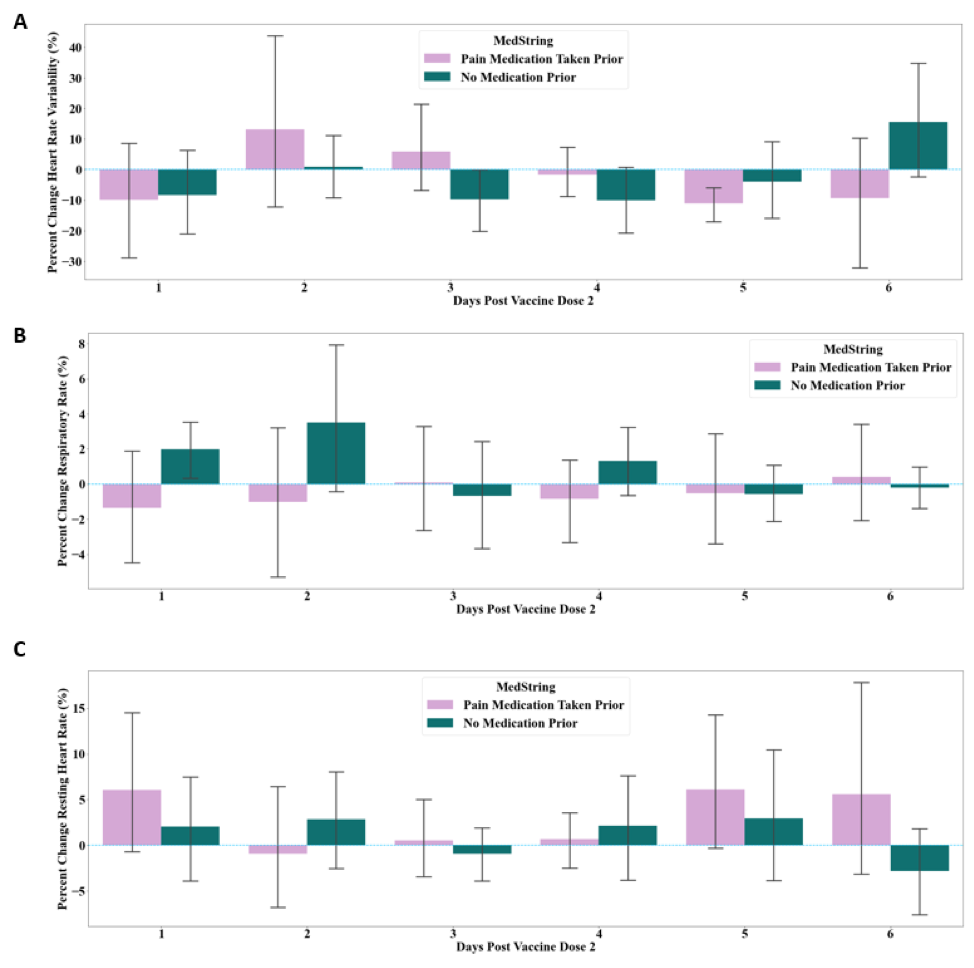

Supplement: Multimedia Appendix 4 [file formative_v5i8e28568_app4.png]

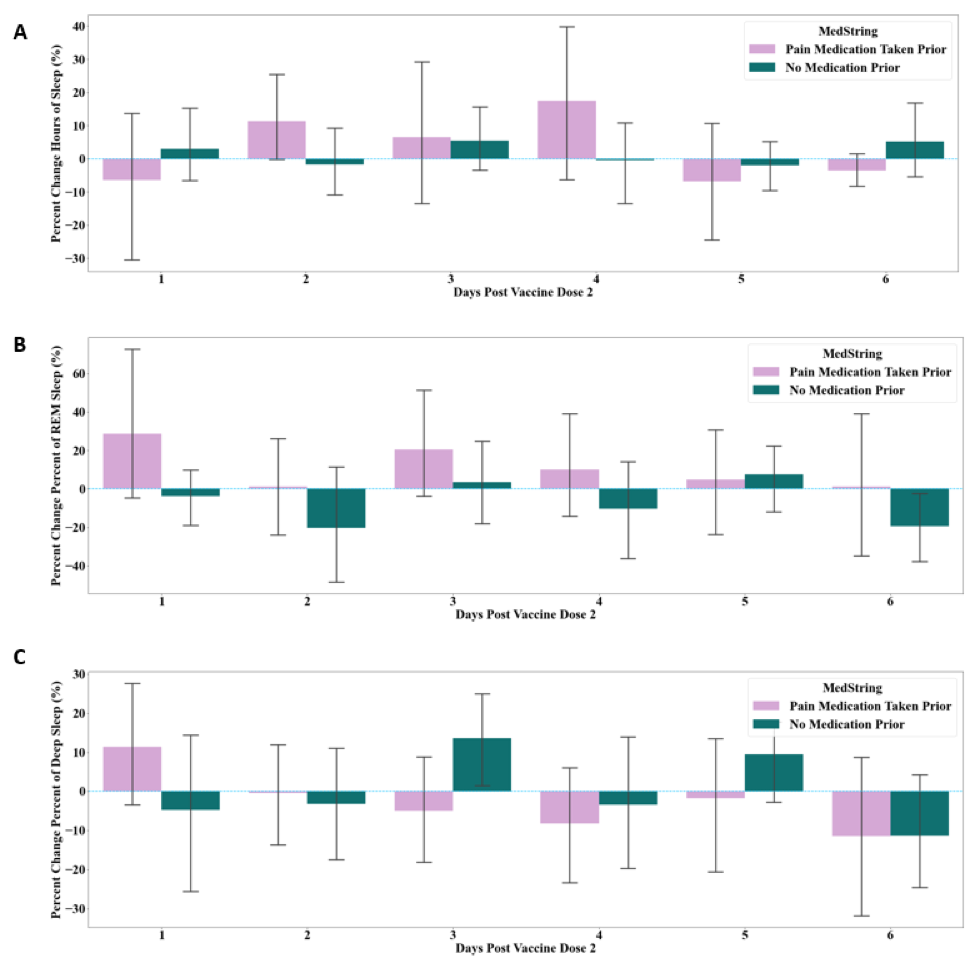

Supplement: Multimedia Appendix 5 [file formative_v5i8e28568_app5.png]
